# Tissue Expansion after Non-Skin-Sparing Mastectomy: A Comparative Study of Expansion Courses of Prepectoral and Subpectoral Tissue Expander Placement with Acellular Dermal Matrix

**DOI:** 10.3390/jcm10194502

**Published:** 2021-09-29

**Authors:** Daiwon Jun, Jin Kwan Kim, Byung Yeun Kwon, Young Jin Kim, Ji Young Rhu, Kwan Ho Lee, Jung Ho Lee

**Affiliations:** 1 Department of Plastic and Reconstructive Surgery, Bucheon St. Mary’s Hospital, College of Medicine, The Catholic University of Korea, Seoul 07345, Korea; jundw430@gmail.com (D.J.); jinkwan30@naver.com (J.K.K.); nebyung@naver.com (B.Y.K.); psyjkim@catholic.ac.kr (Y.J.K.); 2 Department of Surgery, Bucheon St. Mary’s Hospital, College of Medicine, The Catholic University of Korea, Seoul 07345, Korea; jyses82@naver.com (J.Y.R.); lkhdotcome@naver.com (K.H.L.)

**Keywords:** breast neoplasms, mammaplasty, tissue expansion

## Abstract

Although skin- or nipple-sparing mastectomy has been popular in the treatment of breast cancer, the radical excision of breast tissue is unavoidable in certain circumstances. However, the ability of an acellular dermal matrix (ADM) to expand remains questionable, and this situation may further hinder tissue expansion. From October 2017 to January 2020, patients who underwent immediate breast reconstruction with tissue expander placement using ADM whose initial fill volume was less than 50 mL were retrospectively reviewed. The primary outcomes were the number of visits and number of days required to complete the expansion, and the secondary outcomes were the amount of postoperative expansions, expander fill ratio and expander volume. Between the prepectoral group (*n* = 26) and subpectoral group (*n* = 39), the mean number of days (81.46 days versus 88.64 days, *p* = 0.365) and mean number of visits (5.08 versus 5.69, *p* = 0.91) required to complete expansion exhibited no significant differences. Additionally, there were no significant differences in the mean amount of postoperative expansion (314.23 mL versus 315.38 mL, *p* = 0.950), the mean final volume (353.08 mL versus 339.62 mL, *p* = 0.481) or the mean final volume ratio (0.89 versus 0.86, *p* = 0.35) between the two groups. Therefore, we suggest that prepectoral tissue expander placement after conventional mastectomy can be a valid option.

## 1. Introduction

The reconstruction of the breast after mastectomy remains one of the major topics in plastic surgery [1]. The tendency to choose prosthetic over autologous breast reconstruction has been gaining momentum because of its simplicity, reliability and rapid recovery after surgery [2]. From a historical perspective, subpectoral placement has been the chosen method for the majority of prosthetic device placements [3]. This procedure involves the devices being placed under the pectoralis major muscle, wherein the lower pole is covered with or without an acellular dermal matrix (ADM). Such a procedure enables the pectoralis major muscle to act as a soft tissue barrier for prosthetic devices, thus decreasing the possibilities of implant extrusion, rippling or capsular contracture. Although successful, problems remain regarding animation deformity and muscle spasms that can occur from the pectoralis major muscle [4]. Furthermore, patients undergoing radiotherapy have been shown to suffer a higher risk of implant displacement due to fibrosis of the pectoralis major muscle [5,6].

As an alternative, the prepectoral placement of prosthetic devices has been proposed. This method may eliminate the risks of breast deformity and/or implant displacement caused by muscle actions. Additionally, an improvement in postoperative pain caused by submuscular dissection has been reported [7,8,9]. A recent study also reported that compared to partial subpectoral reconstruction, postoperative complications such as seroma, hematoma and animation deformity were significantly higher in prepectoral reconstruction [10].

Innovations and advancements in breast implants, autologous fat grafting, the use of ADM and the acknowledgment of skin-sparing or nipple-sparing mastectomy have accelerated this trend [11,12]. Based on its advantages, the introduction of ADM has set a monumental landmark in the use of prosthetic breast reconstruction [13], including the reinforcement of soft tissue coverage in skin flaps, the segregation of prosthetic devices, a reduction in the inflammatory response and the minimization of the risk of capsular contracture [14,15].

Although skin-sparing mastectomy has become a standard procedure, the radical excision of breast skin is unavoidable in certain circumstances. In that case, due to the reduction in the skin envelope, the initial fill volume of the tissue expander is greatly reduced. Meanwhile, the ability of ADM to expand remains questionable, and this situation may further hinder tissue expansion [16]. Specifically, it is expected that expanders placed in the prepectoral plane will face greater limitations in expansion compared with those placed in the subpectoral plane, wherein the muscular tissue yields a better capability to stretch.

The aim of this study was to compare expansion courses between the prepectoral group and the subpectoral group after non-skin-sparing mastectomy, wherein the initial fill volume of the expander was limited (less than 50 mL). By using the number of visits and number of days required to complete expansion as the primary outcomes, we compared two groups of patients who underwent staged breast reconstruction using ADM. In addition, secondary outcomes were compared, including the mean amount of postoperative expansion (the total amount of infusions performed during the office visits), the expander fill ratio (the cumulative expansion volume divided by the expander volume) and the mean expander volume.

## 2. Materials and Methods

**Figure 1:**
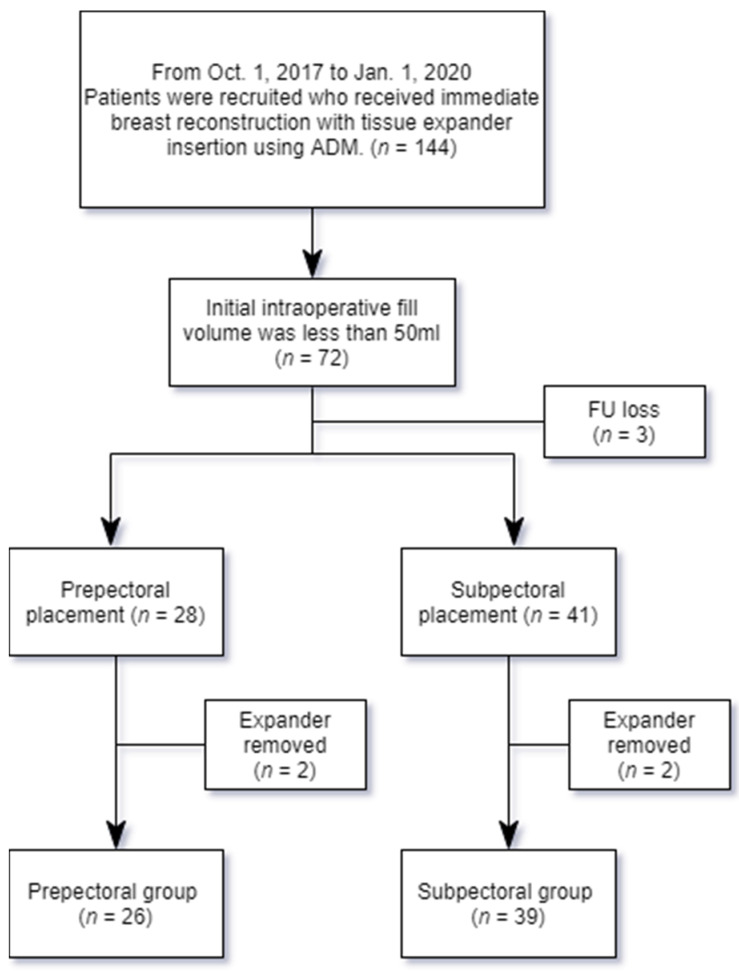
A diagram of the patient recruitment process.

A total of 144 patients who received immediate breast reconstruction with tissue expander placements using ADM from October 2017 to January 2020 were retrospectively reviewed. Among them, a group of 72 patients who had initial fill volumes of less than 50 mL was recruited for this study. Three patients were lost during the follow-up period, and four patients were excluded due to expander removal. Finally, 65 patients who were able to complete expansion were included in the evaluation. The expansion courses were subsequently compared between the prepectoral group (*n* = 26) and the subpectoral group (*n* = 39) (Figure 1). The type of mastectomy performed was either total mastectomy or modified radical mastectomy. Every patient had unilateral breast reconstruction. The inclusion criteria for prepectoral expander placement were as follows: (1) viable skin flap perfusion on physical examination (color and marginal bleeding) or indocyanine green fluorescence angiography, and (2) a thickness of the skin flap greater than 10 mm.

### 2.1. Surgical Techniques

**Figure 2:**
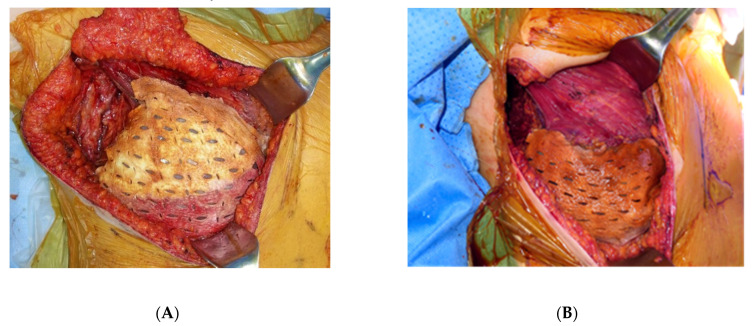
Intraoperative photographs of prepectoral tissue expander placement (**A**) and subpectoral tissue expander placement (**B**).

For patients undergoing prepectoral reconstruction, a deflated tissue expander (Mentor Worldwide LLC, Irvine, CA, USA) was placed in the prepectoral space, and suture tabs were fixed to the pectoralis fascia with #3–0 Vicryl sutures. Inflation of the expander was performed via the maximal infusion of saline until mastectomy wound closure was possible. The volume of expansion in the operating room was defined as the “initial fill volume.” ADM (MegaDerm^®^; L&C Bio Inc, Seoul, Korea), with a thickness of 1 to 2.5 mm, was fenestrated and irrigated prior to insertion. The entire anterior surface of the expander was covered with ADM in vivo, with a basement membrane oriented toward the device. The ADM was sutured to the pectoralis fascia with #3–0 Vicryl sutures (Figure 2A). One or two drains were inserted around the space between the ADM and the mastectomy skin flap, including the axilla, if necessary.

For patients undergoing subpectoral reconstruction, a subpectoral pocket was created by detaching the inferomedial, inferior and lateral borders of the pectoralis major muscle. After placing the expander under the subpectoral pocket, the suture tabs of the tissue expander were sutured to the periosteum of the ribs with #3–0 Vicryl sutures. The lower pole of the expander, which was not covered by the pectoralis major muscle, was covered with ADM (Figure 2B). The upper border of the ADM was sutured to the pectoralis muscle with #3–0 Vicryl sutures, and the lower border of the ADM was sutured to the periosteum of the ribs with #3–0 Vicryl sutures. The initial expansion was performed in the same manner as in the prepectoral reconstruction. One or two drains were inserted around the space between the ADM and the mastectomy skin flap, including the axilla, if necessary.

Expansions were initiated two weeks after surgery in both groups. Each patient visited the office every one or two weeks. At each visit, saline infusion was performed until tightness in the skin flap was observed or if patient discomfort was detected.

### 2.2. Postoperative Complications

Out of the 72 patients who had initial fill volumes of less than 50 mL, three patients were lost during the follow-up period. The complication rate between the prepectoral group (*n* = 28) and the subpectoral group (*n* = 41) was evaluated. Depending on the severity and the required treatment, complications were categorized as either major or minor. Major infection was defined as a culture-proven infection requiring intravenous antibiotic administration or surgical intervention. Major skin necrosis was defined as a full thickness disruption requiring surgical repair. Major seroma was defined as a condition requiring drainage or aspiration. Minor infection was clinically diagnosed and required the administration of oral antibiotics. Minor skin necrosis was defined as superficial wound necrosis that could be managed with the aid of dressings. Minor seroma was defined as a small amount of fluid collection wherein no intervention was required.

### 2.3. Statistical Analysis

All the analyses were performed using IBM SPSS Version 23.0 (IBM Corp., Armonk, NY, USA). Normality was determined by the Shapiro–Wilk test. Normally distributed factors were compared via t tests, and nonnormally distributed variables were evaluated using the Mann–Whitney test. The chi-square test and Fisher’s exact test were used to compare categorical variables. A probability of less than 5% (*p* < 0.05) was used to determine statistical significance.

## 3. Results

### 3.1. Patient Characteristics

Preoperatively, the mean body mass index (BMI) was significantly larger in the prepectoral group. However, a significant difference was not observed in age or history of neoadjuvant chemotherapy, adjuvant chemotherapy or radiotherapy. Patient factors, including diabetes and smoking history, also exhibited no significant differences. Additionally, ductal cell carcinoma in situ (DCIS) was the most common tumor stage in the prepectoral group, whereas stage I and stage II tumors were predominant in the subpectoral group (Table 1).

### 3.2. Perioperative Details

The weight of the excised breast tissue, the area of the excised skin ellipse and the vertical height of the excised skin ellipse demonstrated no statistically significant differences. Total mastectomies were found to be predominant in both groups. The most commonly selected tissue expanders were 350 mL in both groups. However, the area of ADM coverage was larger in the prepectoral group, and the operation time was significantly longer in the subpectoral group (Table 2).

### 3.3. Expansion Outcomes

Table 3 demonstrates the primary and secondary outcomes of this study. Primary outcomes, including the number of visits and the total time period required for the completion of the expansion, did not demonstrate significant differences. Secondary outcomes, including the mean amount of postoperative expansion, mean final volume and mean final volume ratio, also did not demonstrate statistically significant differences.

Table 4, Table 5 and Table 6 demonstrate the expansion courses in detail. Only the intraoperative infusions and the first infusions performed during the office visits were significantly greater in the prepectoral group; no differences were found in the following expansions (Table 4). The prepectoral group had a significantly greater mean expander volume intraoperatively and with the 1st, 2nd and 3rd expansions; however, no differences were found in the following expansions (Table 5). The prepectoral group also had a significantly greater intraoperative expander fill ratio, as well as with the 1st, 2nd and 3rd expansions. No differences were found in the following expansions (Table 6).

### 3.4. Postoperative Complications

No significant difference was found in the overall complication rate between the two groups (Table 7). In each group, two patients received expander removal due to infection.

## 4. Discussion

Breast reconstruction using prosthetic devices has been a primary reconstruction option for decades due to its relative simplicity and rapid recovery compared to autologous reconstruction [17]. Prosthetic breast reconstruction can be performed in one- or two-stage operations. The one-stage (direct-to-implant) approach can only be performed in carefully selected patients who have adequate soft tissue coverage. More than 80% of prosthetic breast reconstructions are performed in two-stage operations due to predictable outcomes and a required time period for pathological confirmation [18].

The revolution in prosthetic devices and the introduction of ADM have popularized the use of prepectoral breast reconstruction. As an alternative to subpectoral placement, prepectoral placement can provide ideal device positioning and eliminate pectoralis major muscle spasms and animation deformities [19]. The use of ADM facilitates the control of the mastectomy space and prosthetic devices; thus, improved aesthetic outcomes can be achieved [13]. ADM is known to minimize fibrosis and promote vascularization in the periprosthetic environment [20], and it also has protective effects regarding both capsular contracture and post-radiotherapy complications [21].

Approaches to mastectomy have evolved in parallel with advancements in prosthetic devices. To preserve the breast skin envelope and facilitate breast reconstruction, modifications in the mastectomy technique have been vigorously sought. Such efforts have proven to provide greater patient satisfaction and equivalent overall survival [22,23]. Therefore, modern mastectomy techniques, i.e., skin-sparing mastectomy (SSM) and nipple-sparing mastectomy (NSM), have become widely accepted [24,25,26]. However, skin preservation is not always possible. In certain situations, such as the proximity of tumors to the skin or skin involvement with the tumor, the radical excision of breast skin is inevitable.

In breast reconstruction after non-skin-sparing mastectomy, the initial fill volume of the expander can be limited due to skin shortages. In this situation, the size of the ADM overlying the deflated expander will be smaller, and the prepectoral pocket created by the ADM will also be finite. For instance, the mean initial fill volume of the prepectoral group in our study was significantly less than that in other studies of prepectoral expansion (41.5 mL versus 223.4 mL by Zhu et al.; 41.5 mL versus 372 mL by Wormer et al.) [27,28]. In addition, the amount of ADM required to create a prepectoral pocket was significantly less in our study than in other studies (141.53 cm2 versus 320 cm2 by Wormer et al.) [27]. For that reason, the ability of ADM to expand is of importance in prepectoral breast reconstruction after non-skin-sparing mastectomy.

The elastic potential of ADM has been demonstrated in various fields of medicine. In the repair of abdominal hernias, ADM has maintained its integrity in more than 95% of cases [29]. However, in the repair of bladder and vaginal suspensions, only 50% were able to achieve the desired result [30]. The ability of ADM to stretch is dependent on various factors, such as the recipient environment and the manner of application (i.e., inlay versus onlay or dermal side up versus dermal side down) [31]. When assuming the environment of the recipient site and the manner of application, the properties of ADM used in abdominal hernias are comparable to those of ADM used in breast reconstruction. Only one study has been conducted to evaluate the elastic properties of ADM used in breast reconstruction. Yang et al. reported that although inserted ADM is resistant to expansion, the pectoralis major muscle and surrounding tissues compensate for the amount of expansion [16]. The above findings suggest that the expansion capability of prepectoral tissue expanders is expected to encounter greater limitations compared to subpectoral tissue expanders. However, our findings suggest that the number of visits and the number of days required to complete expansion did not significantly differ between the two groups. There were significant differences in expander volume and expander fill ratio during the early phase of expansion (i.e., intraoperative to the 3rd postoperative visit) between the groups. However, this can be interpreted by the fact that the differences in the initial fillings were based on higher BMI values in the prepectoral group at baseline. Due to the inclusion criteria, patients with abundant soft tissue were recruited for prepectoral expander placement. Regarding the amount of expansion performed during office visits, only the first infusion was significantly greater in the prepectoral group.

**Figure 3:**
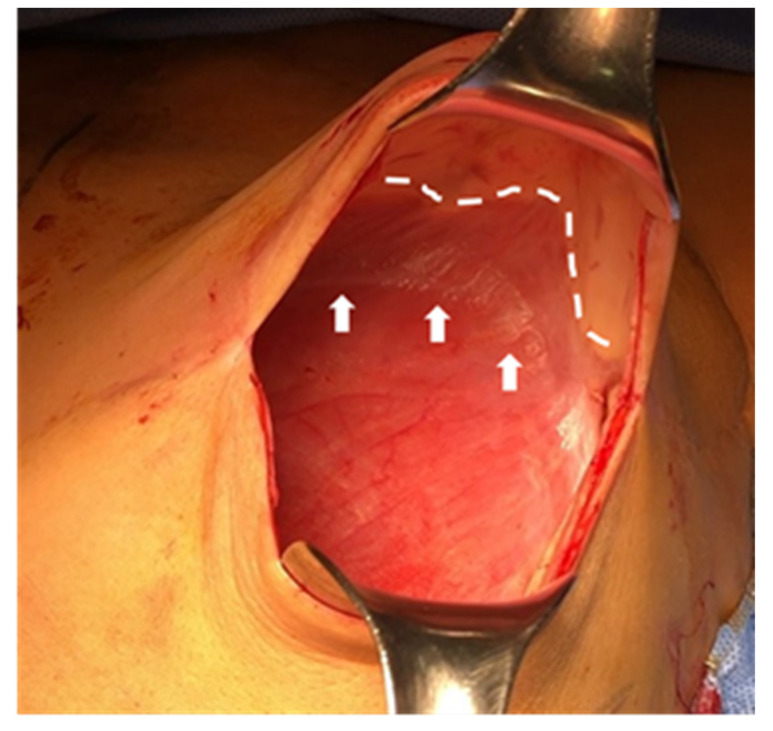
Intraoperative photograph of elongated capsule formation between the acellular dermal matrix (ADM) and the pectoralis muscle fascia. The margin of the pectoralis muscle fascia is indicated with arrows (↑), and the margin of the ADM is indicated by the dashed line.

After interpreting the expansion courses, we postulated two possible explanations. First, although questionable, the lack of ability of ADM to stretch is not significant enough to limit expansion. Second, elongation of the scar tissue that formed between the ADM and native tissue (pectoralis fascia) may have compensated for the limitation of ADM stretching. The authors were able to observe a number of elongated capsules between the ADM and the pectoralis muscle fascia during the 2nd stage of the operation in prepectoral tissue expansion cases (Figure 3).

Our study was confined to the use of thick ADM. Thus, the impact of the thickness of the ADM on expansion was not considered. In addition, future research regarding synthetic mesh-assisted breast reconstruction is warranted. A recent study has demonstrated that in partial subpectoral implant placement, polypropylene mesh-assisted reconstruction showed a significantly lower complication rate compared to ADM-assisted reconstruction [32]. Furthermore, the study design was retrospective in nature. Therefore, it is not free from selection bias, wherein patients with relatively healthy skin flaps were included in the prepectoral group. Finally, due to the relatively short period of the follow-up, long-term postoperative complications, such as animation deformities and capsular contracture, were not considered.

## 5. Conclusions

ADM has become an essential component in breast reconstruction. However, the ability of ADM to stretch is relatively inferior to that of native tissue. In this study, we compared the expansion courses of prepectoral and subpectoral groups with initial fill volumes less than 50 mL. The prepectoral group had a greater mean expander volume and expander fill ratio in the early period of expansion. However, no differences were found in the number of visits or days required for the completion of the expansion. These findings indicate that ADM does not hinder breast tissue expansion. Accounting for the multiple advantages of prepectoral expander placement, i.e., the ease of operation, decreased animation deformity, less pain and improved aesthetic outcomes, prepectoral tissue expander placement can be performed in conventional mastectomy patients even when only a limited space is allowed for expansion.

## Figures and Tables

**Table 1:** Summary of characteristics of patients in the subpectoral and prepectoral groups.

	Subpectoral	Prepectoral	*p*-Value
No. of patients	39	26	
Mean age ± SD, years	49.03 ± 6.88	47.69 ± 8.31	0.484
Mean BMI ± SD, kg/m^2^	21.71 ± 2.76	23.60 ± 2.19	0.005
Neoadjuvant chemotherapy	4	3	0.87
Adjuvant chemotherapy	26	17	0.916
Adjuvant radiotherapy	10	11	0.164
Smoking	1	0	1.000
Diabetes	3	5	0.250
Tumor stage (%)			
DCIS	11 (28%)	11 (42%)	
Stage I	12 (31%)	6 (23%)	
Stage II	12 (31%)	6 (23%)	
Stage III	3 (8%)	3 (12%)	
Prophylactic	1 (3%)	0 (0%)	

BMI: body mass index; DCIS: ductal carcinoma in situ; SD: standard deviations.

**Table 2:** Perioperative details of patients in the subpectoral and prepectoral groups.

	Subpectoral	Prepectoral	*p*-Value
No. of patients	39	26	
Mastectomy specimen weight (g)	344.28	425.81	0.065
Skin ellipse area (cm^2^)	106.18	103.2	0.763
Vertical height of skin ellipse (cm)	7.05	6.68	0.427
Mastectomy type (%)			
Total mastectomy	29 (74%)	17 (65%)	
Modified radical mastectomy	10 (26%)	9 (35%)	
Expander size (%)			
250 mL	0 (0%)	0 (0%)	
275 mL	2 (5%)	1 (4%)	
350 mL	19 (49%)	14 (54%)	
450 mL	16 (41%)	9 (35%)	
550 mL	2 (5%)	2 (8%)	
ADM * surface area (cm^2^)	88.69	141.53	<0.001
Operation time (min)	76.56	58.46	<0.001

ADM *: acellular dermal matrix.

**Table 3:** Summary of the primary and secondary outcomes.

	Subpectoral	Prepectoral	*p*-Value
No. of patients	39	26	
Mean time to completion (days)	88.64	81.46	0.365
Mean no. of visits for completion	5.69	5.08	0.91
Mean amount of postoperative expansion (mL)	315.38	314.23	0.950
Mean final volume (mL)	339.62	353.08	0.481
Mean final volume ratio	0.86	0.89	0.35

**Table 4:** Comparison of the expansion process between the subpectoral and prepectoral groups according to the mean amount of expansion per visit.

Mean Amount of Expansion Per Visit (mL)	Subpectoral (*n* = 39, %)	Prepectoral (*n* = 26, %)	*p*-Value
Intraoperative	27.9 (100%)	41.5 (100%)	0.011
1st postoperative	57.1 (100%)	68.8 (100%)	0.014
2nd postoperative	65.5 (100%)	71.7 (100%)	0.236
3rd postoperative	63.1 (100%)	63.6 (96%)	0.955
4th postoperative	60.3 (100%)	55.6 (96%)	0.337
5th postoperative	49.2 (82%)	55 (73%)	0.582
6th postoperative	41.2 (43%)	38.2 (42%)	0.675
7th postoperative	32.5 (15%)	35 (7%)	1.00

**Table 5:** Comparison of the expansion process between the subpectoral and prepectoral groups according to the mean expander volume.

Mean Expander Volume (mL)	Subpectoral (*n* = 39)	Prepectoral (*n* = 26)	*p*-Value
Intraoperative	27.9	41.5	0.011
1st postoperative	85	111.1	0.014
2nd postoperative	150.5	183.8	0.030
3rd postoperative	213.5	246.9	0.037
4th postoperative	275.4	300.6	0.106
5th postoperative	319.5	350.8	0.208
6th postoperative	345.3	386.3	0.378
7th postoperative	366.6	385	0.429

**Table 6:** Comparison of the expansion process between the subpectoral and prepectoral groups according to the expander fill ratio.

Expander Fill Ratio	Subpectoral (*n* = 39)	Prepectoral (*n* = 26)	*p*-Value
Intraoperative	0.07	0.11	0.013
1st postoperative	0.21	0.28	0.022
2nd postoperative	0.38	0.46	0.026
3rd postoperative	0.54	0.63	0.036
4th postoperative	0.69	0.76	0.122
5th postoperative	0.85	0.85	0.178
6th postoperative	0.87	0.96	0.111
7th postoperative	0.85	0.96	0.071

**Table 7:** Comparison of postoperative complications between the subpectoral and prepectoral groups.

Complication	Subpectoral	Prepectoral	*p*-Value
No. of expanders	41	28	
Any complication	12	9	0.799
Major complications	9	8	
Infection	5	5	
Skin necrosis	3	2	
Seroma	1	1	
Minor complications	3	1	
Infection	2	1	
Skin necrosis	1	0	
Seroma	0	0	

## Data Availability

The data presented in this study are available on request from the corresponding author. The data are not publicly available due to privacy or ethical issues.
